# Clinical Evaluation of a Stool‐Based Molecular Point‐of‐Care Test Kit for Noninvasive Detection of *Helicobacter pylori* Infection and Clarithromycin Resistance

**DOI:** 10.1155/cjgh/4505059

**Published:** 2026-10-04

**Authors:** Momoko Tsuda, Yoshiyuki Watanabe, Kimitoshi Kubo, Mototsugu Kato

**Affiliations:** ^1^ Department of Gastroenterology National Hospital Organization Hakodate Medical Center Hakodate Japan; ^2^ Department of Gastroenterology Public Interest Foundation Hokkaido Cancer Society Sapporo Japan; ^3^ Department of Internal Medicine Kawasaki Rinko General Hospital Kawasaki Japan; ^4^ Department of Internal Medicine Division of Gastroenterology St. Marianna University School of Medicine Kawasaki Japan marianna-u.ac.jp

**Keywords:** *H. pylori*, intragastric fluid, microbial sensitivity tests, point-of-care testing, polymerase chain reaction, stool

## Abstract

**Aim:**

Although molecular point‐of‐care testing using intragastric fluid has improved the speed and accuracy of *Helicobacter pylori (H. pylori)* diagnosis and clarithromycin (CAM) resistance detection, it still requires endoscopy, which limits its application in routine screening and settings where invasive procedures cannot be performed. We aimed to evaluate the clinical performance of the Smart Gene™ molecular point‐of‐care testing kit using stool specimens for *H. pylori* infection and CAM resistance detection.

**Methods:**

We used stool specimens from 75 patients with suspected *H. pylori* infection. All participants underwent conventional diagnostic tests (urea breath test, stool antigen test, culture, and real‐time polymerase chain reaction). CAM resistance–associated mutations (Positions 2142 and 2143 in the 23S rRNA gene) were analyzed using pyrosequencing.

**Results:**

The stool‐based Smart Gene kit demonstrated a sensitivity of 96.1% and specificity of 100% for *H. pylori* detection. The sensitivity and specificity for detecting CAM resistance were 100% and 97.0%, respectively, compared to antibiotic susceptibility testing. High concordance was observed between the intragastric polymerase chain reaction and culture results despite the lower stool DNA concentration. The results were obtained within approximately 1 h.

**Conclusions:**

The Smart Gene™ stool kit offers a rapid, accurate, and noninvasive alternative for diagnosing *H. pylori* infection and CAM resistance. Its diagnostic performance is comparable to that of the gold‐standard invasive methods while offering practical advantages for outpatient use, mass screening, and nonendoscopy‐feasible populations. This approach increases early *H. pylori* detection and improves eradication outcomes and contributes to more effective gastric cancer prevention strategies (approval number: R4‐0426001).

## 1. Introduction

*Helicobacter pylori* (*H. pylori*) is a Gram‐negative bacterium that causes chronic inflammation and disrupts the gastric mucosal barrier, leading to peptic ulcer disease and gastric cancer [1]. As *H. pylori* eradication lowers the risk of gastric cancer, the International Agency for Research on Cancer and the Cancer Research Agency of the World Health Organization strongly recommend implementing *H. pylori* eradication therapy as a global preventive method for gastric cancer [2–5].

In Japan, since national insurance coverage for “*H. pylori*‐infected gastritis,” one of the diseases targeted for eradication therapy, started in 2013, *H. pylori* eradication has significantly increased, and deaths due to gastric cancer are gradually decreasing [4, 6]. However, to further reduce the risk of gastric cancer by improving eradication rates, the Japanese Society for *Helicobacter* Research guidelines recommend *H. pylori* eradication therapy as the primary prevention strategy for gastric cancer after antibiotic susceptibility testing (AST). *H. pylori* eradication is prioritized as a primary gastric cancer prevention procedure through AST [7]. Since clarithromycin (CAM) resistance compromises the efficacy of *H. pylori* eradication therapy and has become a leading cause of treatment failure, addressing CAM resistance is essential for improving treatment outcomes [8, 9].

CAM resistance in *H. pylori* is primarily caused by point mutations at Positions 2142 and 2143 in the domain V region of the 23S rRNA gene [10]. CAM resistance‐associated mutations can be detected using intragastric fluid samples, offering a molecular‐based alternative to conventional culture methods that require gastric mucosal biopsy [11]. Although this approach helps shorten testing time, it still requires endoscopy, making the procedure invasive and less suitable for routine clinical use, particularly in populations such as older adults or children who cannot undergo endoscopy.

Smart Gene™ is a fully automated genetic analyzer designed for point‐of‐care testing (POCT). This system is based on the QProbe polymerase chain reaction (PCR) method, which detects pathogenic microorganisms and genomic mutations associated with antibiotic resistance [12]. Smart Gene™ developed a kit that can automatically detect *H. pylori* and CAM resistance simultaneously [13]. Smart Gene™ can use intragastric liquid as a testing specimen to diagnose *H. pylori* infection and provide accurate results within an hour. However, the *H. pylori* kit requires an endoscopic examination to collect intragastric fluid, making it difficult to use in individuals who cannot undergo endoscopy, such as older adults and children.

Despite the advantages of the Smart Gene™ system in providing rapid results for *H. pylori* diagnosis and CAM resistance, the system cannot be considered truly noninvasive as long as obtaining intragastric fluid requires endoscopic examination, and this remains a barrier to routine use in clinical practice.

To explore the limitations of the current diagnostic approaches, we need more time‐efficient, patient‐friendly, and less invasive alternatives for diagnosing *H. pylori* infection and detecting CAM resistance that do not rely on endoscopy. An accurate diagnosis that does not require specialized equipment or invasive procedures would enable broader implementation, improve patient accessibility and comfort, and contribute to earlier diagnoses and more effective eradication strategies at the population level. Recent reports on antibiotic resistance in *H. pylori* have underscored the clinical relevance of molecular AST in selective eradication regimens. In particular, stool‐based molecular diagnostics have emerged as a noninvasive and promising approach; however, the lack of widely accessible testing platforms and the need for further clinical validation remain significant challenges [14]. To address this need, we evaluated the clinical performance of the Smart Gene™ molecular POCT kit using stool specimens for the detection of *H. pylori* infection and CAM resistance [15, 16].

We aimed to determine whether stool sample testing could offer diagnostic accuracy comparable to that of conventional invasive methods while improving accessibility for routine clinical use. This paper presents the clinical performance of the Smart Gene™ molecular POCT kit using stool specimens as a practical, noninvasive alternative for detecting *H. pylori* infection and CAM resistance, thereby resolving current limitations of invasiveness and long testing time in conventional diagnostic approaches. Through this study, we aim to demonstrate that stool sample molecular testing can offer a practical and noninvasive alternative to current methods and support broader clinical use for outpatients and mass screening for prioritized primary gastric cancer prevention procedures.

To the best of our knowledge, this is the first study to evaluate the Smart Gene™ system—previously validated only for use with intragastric fluid—using stool specimens, with direct, within‐subject comparison against five independent reference and comparator methods (urea breath test [UBT], stool antigen test [SAT], culture, and real‐time PCR of both stool and intragastric fluid) in the same cohort. Unlike previous stool‐based PCR studies, which have generally focused on either infection detection or CAM resistance alone, our study simultaneously evaluated *H. pylori* infection status and CAM resistance—including heterozygous (hetero‐resistant) genotypes—within a single rapid assay completed in approximately 1 h. These findings demonstrate not only the diagnostic noninferiority of stool‐based testing but also its practical feasibility for point‐of‐care implementation in outpatient and mass‐screening settings.

## 2. Materials and Methods

### 2.1. Patient Enrollment and Conventional *H. pylori* Testing

To evaluate the diagnostic performance of a stool‐based method for *H. pylori* detection (Smart Gene™ stool kit), we conducted a prospective diagnostic accuracy study in patients with suspected *H. pylori* infection at our institution between August 2021 and November 2022. A sample size calculation was performed assuming a high concordance between the stool‐based assay and reference methods. Using an expected kappa of 0.80, a precision of 0.05, and a Z‐score of 1.96, the minimum required sample size was estimated as 62 patients, based on the formula *n* = *Z* ^2^(1 − *κ*)^2^/*d* ^2^. A total of 75 patients were included, exceeding the required number to ensure adequate precision for noninferiority assessment (Figure 1). In addition to collecting stool samples, all 75 participants underwent conventional tests, such as the UBI (UBIT tablet, 100 mg; Otsuka Pharmaceutical Co., Ltd., Tokyo, Japan), SAT (Testmate rapid pylori antigen; Wakamoto Pharmaceutical Co., Ltd, Kanagawa, Japan), *H. pylori* culture test (BML Co., Ltd., Tokyo, Japan), and real‐time PCR of intragastric fluid to validate the accuracy of the stool‐based detection method. Culture, concordant with real‐time PCR of intragastric fluid, served as the reference standard for assessing *H. pylori* infection status, and AST served as the reference standard for determining CAM resistance. UBT and SAT, both noninvasive tests already used in routine clinical practice, were additionally included as comparator tests to benchmark the diagnostic performance of the stool‐based Smart Gene™ assay against other clinically available noninvasive testing options. Inclusion criteria included patients scheduled for endoscopic examination with no prior history of *H. pylori* eradication. Exclusion criteria included recent antibiotic use within the previous 4 weeks or inability to provide informed consent. Six patients were receiving PPIs or P‐CABs; however, because PCR‐based assays are not affected by acid‐suppressive therapy and are endorsed by Japanese guidelines for use in such patients, PPI/P‐CAB use was not considered an exclusion criterion.

**FIGURE 1:**
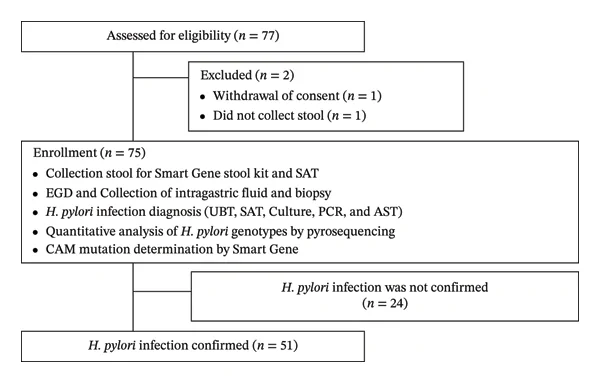
Flowchart of patient enrollment and testing procedures. Of 77 patients assessed for eligibility, two were excluded—one withdrew consent, and one failed to provide a stool sample. The remaining 75 participants underwent stool sample collection for Smart Gene™ testing and SAT, followed by EGD with intragastric fluid and biopsy collection. Diagnostic evaluations included UBT, SAT, culture, intragastric PCR, and AST. CAM resistance–associated mutations were further analyzed via Smart Gene™ and pyrosequencing. EGD, esophagogastroduodenoscopy; SAT, stool antigen test; UBT, urea breath test; PCR, polymerase chain reaction; AST, antibiotic susceptibility testing; CAM, clarithromycin.

### 2.2. Collection of Stool Samples

To ensure suitability for testing, all stool samples were collected under standardized conditions, including the time of collection, storage temperature, and time for transport. We made a “stool collection container” that enabled the patients to collect enough stool for testing, which contains a specially designed liquid for sample transport and preservation. First, the patient opens the lid of the container and finds a stick attached to the lid. They then collect stool samples using the stick by evenly rubbing the surface such that the samples reflect the entire stool condition. The patient then places the stick back in the container and closes the lid. After collecting the samples, patients mail their samples to testing medical institutions that own Smart Gene to perform the test (Figure 2A). The collected “stool collection containers” were stored and transported for no more than 7 days at temperatures below 30°C, or no more than 14 days at temperatures below 8°C, or if this was difficult, frozen at temperatures below −20°C, before being used for testing.

**FIGURE 2:**
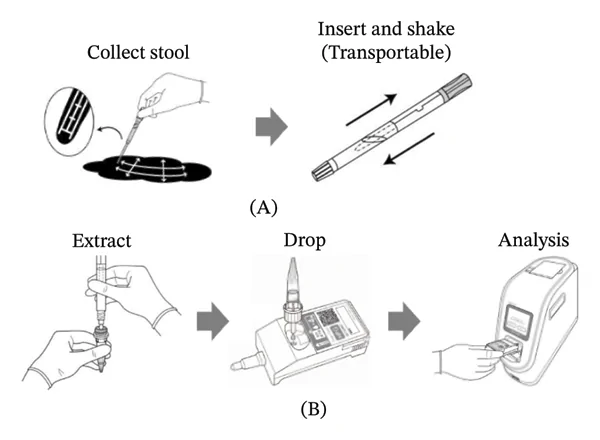
Workflow for stool specimen analysis using the Smart Gene™ molecular POCT kit. (A) Stool collection protocol. Patients collect stool using the provided container, which is shaken after insertion and transported without requiring immediate refrigeration. (B) Laboratory procedure. The stool extract is dropped into the test cartridge and inserted into the Smart Gene™ device for automated DNA extraction, amplification, and mutation detection within approximately 1 h. POCT, point‐of‐care testing.

### 2.3. Gastric Mucosal Biopsy and Intragastric Fluid Sample Collection

To validate the accuracy of the stool‐based detection method, we compared the results with those obtained using conventional diagnostic methods, including *H. pylori* culture and real‐time PCR using intragastric fluid [13]. We collected the patients’ intragastric fluid and performed gastric mucosal biopsy as part of the endoscopic examination procedures, as described previously [11, 13]. Prior to endoscopy, each patient ingested 100 mL of a premedication solution consisting of 80 mg of dimethylpolysiloxane, 1 g of sodium bicarbonate, and 20,000 units of pronase to facilitate mucosal visualization. During the procedure, mucosal biopsies measuring approximately 5 mm were obtained using standard biopsy forceps. We obtained three biopsy samples from the gastric antrum and three from the greater curvature of the gastric body. In this study, these biopsy specimens were all used for culture and AST. Rapid urease testing (RUT) and histological examination were not performed. Because *H. pylori* culture carries an inherent risk of false‐negative results owing to its fastidious growth requirements, this larger number of specimens was allocated to maximize culture sensitivity and to ensure a sufficient bacterial yield for reliable AST. Intragastric fluid was collected through the endoscope’s suction channel directly in to a sample container (MD‐33050; SB‐KAWASUMI LABORATORIES, Inc., Tokyo, Japan). We compared the concordance between the stool‐based method and the established tests currently used in clinical practice.

### 2.4. *H. pylori* AST

To determine the susceptibility of *H. pylori* to CAM, we performed AST using a gastric mucosal biopsy as the conventional diagnostic method. A contract laboratory (BML Co., Ltd., Tokyo, Japan) carried out the procedure. First, we cultured the biopsy samples on Helicobacter‐selective agar (Nissui Pharmaceutical Co., Ltd., Tokyo, Japan) at 37°C for 4 days under microaerophilic conditions (5% O_2_, 15% CO_2_, and 80% N_2_). We then selected three colonies and subcultured them on 5% sheep blood agar (Nippon Becton Dickinson Co., Ltd., Tokyo, Japan). After adjusting the bacterial suspension to a McFarland 1.0 standard in saline, we added 25 μL to 6 mL of Mueller–Hinton broth supplemented with 10% horse serum (Nikken Biological Co., Ltd., Kyoto, Japan). We used the dry plate method developed by Eiken Chemical Co., Ltd. (Tokyo, Japan) to conduct the CAM susceptibility testing, which is based on the broth microdilution method. Finally, we interpreted the minimum inhibitory concentrations (MICs) based on the 1 μg/mL breakpoint criteria defined by the Japan Society of Chemotherapy and the Clinical and Laboratory Standards Institute [17].

### 2.5. Stool‐Based Smart Gene Assay for Detection of *H. pylori* DNA and CAM‐Resistant Mutation

To assess the feasibility of using stool samples with the *H. pylori* molecular POCT kit (Smart Gene; Mizuho Medy Co., Ltd., Tosu, Japan), we evaluated its ability to detect *H. pylori* and CAM resistance–associated mutations. We introduced the liquid stool sample into the Smart Gene™ test cartridge designed for *H. pylori* detection. This system uses PCR amplification combined with QProbe technology to identify *H. pylori* DNA and detect CAM resistance–associated point mutations at nucleotide Positions A2142G and A2143G within the 23S rRNA gene [18]. Smart Gene automatically and simultaneously processed the diagnosis of *H. pylori* infection and detection of CAM resistance–associated mutations and produced the results within an hour (Figure 2B).

### 2.6. DNA Extraction From Stool Samples and Intragastric Fluid

DNA extraction and purification procedures were performed for both stool and intragastric fluid samples. For stool, DNA was extracted from 100 μL of stool collection liquid with the QIAamp DNA Mini Kit (QIAGEN GmbH, Hilden, Germany) and concentrated with the NucleoSpin Gel and PCR Clean‐up (Takara Bio, Kusatsu, Japan) to obtain 20 μL of purified DNA. For intragastric fluid, DNA was extracted from 200 μL of intragastric fluid with the QIAamp DNA Mini Kit to obtain 150 μL of purified DNA.

### 2.7. Quantitative Detection of *H. pylori* 16S rRNA Real‐Time PCR

To compare the outcomes of the different testing methods, we performed real‐time PCR targeting the *H. pylori* 16S rRNA gene using DNA purified from both stool and intragastric fluid samples. We designed the following primer pair for amplification: forward primer CGC‐TAA‐GAG‐ATC‐AGC‐CTA‐TGT‐CC and reverse primer CCG‐TGT‐CTC‐AGT‐TCC‐AGT‐GTG‐T to detect the *H. pylori* 16S rRNA gene. Reactions were carried out on the Thermal Cycler Dice Real‐Time System III instrument (Takara Bio, Kusatsu, Japan) with TB Green Premix Dimer Eraser Perfect Real‐Time Reagent. The PCR protocol conditions were preheating at 95°C for 30 s, followed by 50 cycles of 95°C for 5 s, annealing at 55°C for 30 s, and extension at 72°C for 30 s. Melting curve analysis confirmed the specificity of the amplified products.

### 2.8. Quantitative Analysis of *H. pylori* CAM‐Resistant Mutation Ratio by Pyrosequencing

To assess and determine CAM resistance, we quantified the mutation frequencies at Positions 2142 and 2143 of the *H. pylori* 23S rRNA gene by pyrosequencing following previously reported procedures [11, 13]. We extracted purified DNA from both stool and intragastric fluid samples. PCR amplification with biotin‐labeled primers yielded a 138‐bp product of the 23S rRNA gene. The forward primer was GAG‐GTG‐AAA‐ATT‐CCT‐CCT‐ACC‐CGC‐G, and the reverse primer was GGC‐TCC‐ATA‐AGA‐GCC‐AAA‐GCC‐CTT‐AC. We employed a touchdown PCR approach beginning with denaturation at 94°C for 120 s, followed by annealing at 98°C for 10 s, and extension through a temperature gradient 74°C to 68°C for 30 s. The amplified fragments were subjected to pyrosequencing analysis using the PyroMark Q24 platform (QIAGEN, Valencia, CA, USA) and the sequencing primer ACC‐CGC‐GGC‐AAG‐ACG.

### 2.9. Ethical Approval

This study was approved by the Medical Ethics Committee of the National Hospital Organization Hakodate Medical Center (approval number: R4‐0426001). Written informed consent was obtained from all the participants prior to enrollment.

### 2.10. Statistical Analysis

We assessed the agreement between the Smart Gene results and reference diagnostic methods by calculating Cohen’s kappa values. Confidence intervals (95%) for sensitivity and specificity were estimated using the Clopper–Pearson method, and for Cohen’s kappa using the normal approximation. For comparison of the relative quantities of *H. pylori* DNA between stool and intragastric fluid samples, a paired *t*‐test was performed on log‐transformed data. All data analyses were performed using the R 4.3.1 software program (https://www.r-project.org).

## 3. Results

### 3.1. Patient Enrollment, Prevalence, and Baseline Characteristics

To evaluate the diagnostic performance of a stool‐based method for *H. pylori* detection (Smart Gene™ stool kit), we enrolled 77 patients with suspected *H. pylori* infection at our institution between August 2021 and November 2022. Two patients were excluded because they did not provide consent (*n* = 1) or stool sample (*n* = 1), resulting in 75 patients being enrolled in the study (Figure 1). Using culture and AST as the reference standards, the prevalence of *H. pylori* infection in this cohort was 68.0% (51/75), and the prevalence of CAM resistance among infected patients was 33.3% (17/51).

### 3.2. Comparison of *H. pylori* Infection Diagnosis Using the Smart Gene Stool Kit With Conventional Methods

The results of the Smart Gene stool kit strongly correlated with the results from conventional *H. pylori* diagnostic methods, such as UBT, SAT, culture tests, and real‐time PCR of stool and intragastric fluid (Table 1). The culture test and Smart Gene stool kit (sensitivity of 96.1% [49/51] and specificity of 100% [24/24]) showed the highest concordance rate.

**TABLE 1:** Comparison of *Helicobacter pylori* infection diagnosis using the Smart Gene stool kit and conventional methods.

Control methods	Detection of *H. pylori* DNA by Smart Gene stool kit				
	Positive	Negative	Sensitivity	Specificity	Cohen’s *κ*
UBT					
Positive	47	5	90.4% (79.0%–96.8%)	91.3% (72.0%–98.9%)	0.788 (0.639–0.938)
Negative	2	21			
SAT					
Positive	47	3	94.0% (83.5%–98.7%)	92.0% (74.0%–99.0%)	0.851 (0.726–0.977)
Negative	2	23			
Culture					
Positive	49	2	96.1% (86.5%–99.5%)	100% (85.8%–100%)	0.940 (0.858–1.00)
Negative	0	24			
Real‐time PCR (stool)					
Positive	49	2	96.1% (86.5%–99.5%)	100% (85.8%–100%)	0.940 (0.858–1.00)
Negative	0	24			
Real‐time PCR (intragastric fluid)					
Positive	49	2	96.1% (86.5%–99.5%)	100% (85.8%–100%)	0.940 (0.858–1.00)
Negative	0	24			

*Note:* Data in parentheses are 95% confidence intervals.

Abbreviations: PCR, polymerase chain reaction; SAT, stool antigen test; UBT, urea breath test.

### 3.3. Discrepancies in the Diagnosis of *H. pylori* by Smart Gene

Mismatches between the Smart Gene stool kit and conventional *H. pylori* diagnostic methods occurred in 10 cases (Table 2). In two cases, the Smart Gene stool kit showed false‐negative results.

**TABLE 2:** List of mismatch cases between Smart Gene stool kit and control methods.

Case no.	Control methods					Smart gene stool kit
	UBT	SAT	Culture	Real time PCR (stool)	Real time PCR (intragastric fluid)	
1	+	+	+	+	+	−
2	+	+	+	+	+	−
3	+	−	+	+	+	+
4	+	−	+	+	+	+
5	+	−	−	−	−	−
6	+	−	−	−	−	−
7	+	−	−	−	−	−
8	−	+	+	+	+	+
9	−	+	+	+	+	+
10	−	+	−	−	−	−

Abbreviations: PCR, polymerase chain reaction; SAT, stool antigen test; UBT, urea breath test.

Among these mismatches, three cases showed a positive UBT result but negative culture and PCR results (Table 2, Cases 5–7); the UBT values in these cases were 2.5‰, 2.9‰, and 7.7‰, respectively. Two of these values (2.5‰ and 2.9‰) were close to the assay cutoff (2.5‰), suggesting that these most likely represented false‐positive UBT results rather than true infection. Among the six patients receiving PPI or P‐CAB therapy, three were positive across all tests (UBT, SAT, culture, and PCR) and were confirmed as infected, two were negative across all tests and were confirmed as uninfected, and one patient was UBT‐negative but positive on SAT, culture, and PCR, suggesting that PPI/P‐CAB use may have reduced UBT sensitivity in this case.

### 3.4. Comparison of CAM Resistance Determined by Smart Gene Stool Kit and AST

The Smart Gene™ stool kit demonstrated high diagnostic accuracy in detecting CAM resistance, showing excellent sensitivity 100% (16/16) and specificity 97.0% (32/33) compared to AST (Table 3).

**TABLE 3:** Comparison of CAM resistance determined by Smart Gene stool kit and antibiotic susceptibility testing.

Control method	Detection of CAM resistance mutation using the Smart Gene stool kit				
	Mutation	Wild type	Sensitivity	Specificity	Cohen’s κ
AST					
Resistant	16	0	100% (79.4%–100%)	97.0% (84.2%–99.9%)	0.954 (0.866–1.00)
Sensitive	1	32			

*Note:* Data in parentheses are 95% confidence intervals. CAM, clarithromycin.

Abbreviation: AST, antibiotic susceptibility testing.

Among the 17 cases in which the Smart Gene™ stool kit detected CAM resistance–associated mutations, heterozygous (hetero‐resistant) genotypes were identified in 7 cases (41.2%) by pyrosequencing analysis. One of these seven cases showed a discordant result with AST, in which the isolate was phenotypically determined to be susceptible despite mutation detection.

### 3.5. Comparative Evaluation Results for Intragastric Fluid and Stool Samples

Although the quantity of *H. pylori* DNA in stool samples was approximately 1/1,000 that in intragastric fluid, the diagnostic accuracy for *H. pylori* infection remained comparable between the two samples (Figure 3). Furthermore, the pyrosequencing analysis revealed high concordance between stool and intragastric fluid samples for the detection of CAM resistance–associated mutations (Figure 4).

**FIGURE 3:**
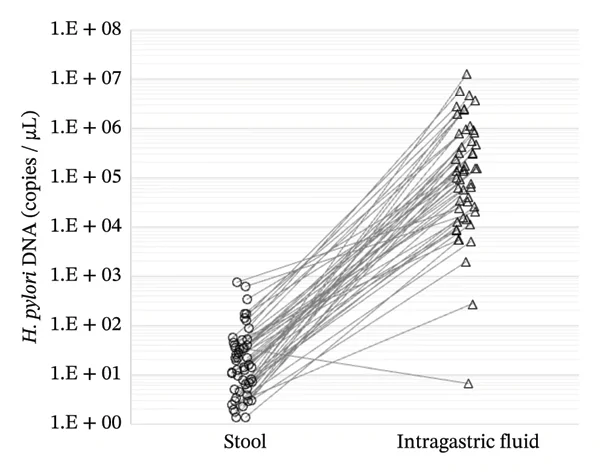
Comparison of the quantity of *Helicobacter pylori* DNA obtained from intragastric fluid and stool specimens. The relative quantity of *H. pylori* DNA extracted from stool was significantly lower than that from intragastric fluid; however, diagnostic performance remained comparable between the two specimens. A paired *t*‐test on log10‐transformed DNA quantities confirmed a statistically significant difference between stool and intragastric fluid (mean difference in log10 copies/μL = −3.79, 95% CI −4.15 to −3.43, *p* < 2.2 × 10^−16^).

**FIGURE 4:**
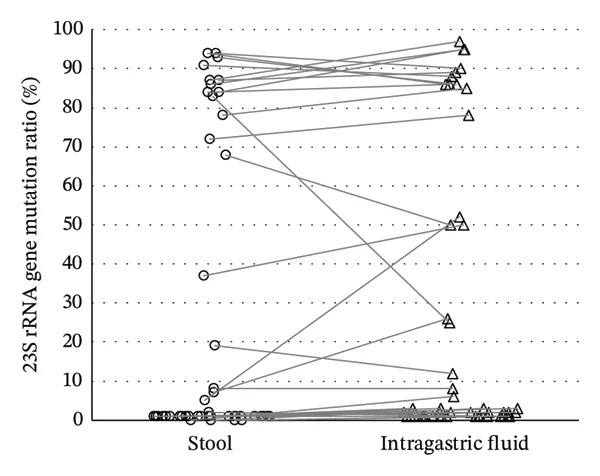
Concordance of CAM resistance–associated mutation detection between intragastric fluid and stool specimens. Pyrosequencing results showed high agreement in the detection of mutations at positions 2142 and 2143 of the 23S rRNA gene between stool and intragastric fluid samples, supporting the reliability of stool‐based molecular testing. CAM, clarithromycin.

## 4. Discussion

The Smart Gene™ stool kit demonstrated strong diagnostic agreement with conventional testing methods for both *H. pylori* detection and CAM resistance, indicating that a noninvasive, stool‐based approach would achieve diagnostic performance comparable to invasive gold‐standard methods and serve as a reliable alternative in clinical settings. The sensitivity and specificity of the kit for *H. pylori* detection were highly reliable compared to those of culture tests and real‐time PCR of intragastric fluid. The kit also achieved high sensitivity and specificity in AST for CAM resistance, suggesting its high potential in clinical practice. These results are consistent with earlier findings, demonstrating its reliability in detecting point mutations in the 23S rRNA gene [13, 19]. Although the authors in [19] employed a droplet digital PCR‐based approach that differs from the QProbe PCR technology used in the Smart Gene™ system, both approaches share the common principle of detecting 23S rRNA point mutations in stool specimens and have similarly demonstrated high concordance with AST, supporting the overall rationale for stool‐based molecular diagnostics of CAM resistance. While prior studies have established the high accuracy of fecal PCR for detecting CAM resistance, our results demonstrated equally high or even superior performance under routine clinical practice. This finding underscores not only the robustness of stool‐based PCR diagnostics but also their practical feasibility in daily clinical practice [20, 21].

Furthermore, heterozygous (hetero‐resistant) genotypes were identified in 7 of the 17 mutation‐positive cases (41.2%) by pyrosequencing in the present study, indicating that the QProbe PCR technology underlying the Smart Gene™ stool kit, as previously demonstrated for other pathogens, is also capable of detecting hetero‐resistant *H. pylori* populations, which are reported to occur in up to 20% of treatment‐naïve patients [12].

Beyond CAM, stool‐based molecular assays have also been applied to detect resistance‐associated mutations for other antibiotics used in *H. pylori* eradication regimens, including levofloxacin, metronidazole, amoxicillin, and tetracycline, suggesting that this noninvasive platform could be extended in the future to provide a more comprehensive antimicrobial susceptibility profile [22]. As the correlation between genotypic mutation and phenotypic resistance has not yet been fully validated for all antimicrobial classes, further studies are warranted to establish the clinical utility of this expanded approach.

Compared with UBT and SAT, the Smart Gene™ stool assay provides an additional advantage in that it enables simultaneous CAM resistance determination and can be reliably used in patients receiving PPI or P‐CAB therapy, for whom UBT sensitivity may be reduced.

Stool‐based testing offers diagnostic accuracy comparable to and, in some cases, exceeding that of gastric specimens (intragastric fluid) in detecting *H. pylori* infection and CAM resistance. Stool‐based PCR testing provides a noninvasive, patient‐friendly alternative to intragastric fluid or biopsy specimens, which typically require endoscopy [23]. While intragastric fluid can be used to detect CAM resistance–associated mutations, this method still requires endoscopy, making it unsuitable for certain populations, such as children, older individuals, or those unwilling to undergo invasive procedures, whereas stool collection is simple, painless, and feasible across all age groups [24]. Additionally, stool samples represent the full spectrum of gastrointestinal colonization that can be missed in localized biopsy specimens [25]. Since biopsy specimens reflect only a limited part of the gastric mucosa, they would underestimate the presence of resistant strains [26].

The combination of the Smart Gene™ system with stool specimens enables a rapid, safe, and noninvasive diagnostic approach that has the potential to transform *H. pylori* management, facilitating effective outpatient treatment and large‐scale screening without the need for endoscopy. Compared to conventional tests, stool‐based Smart Gene testing is more practical and better suited for widespread clinical use. While maintaining comparable diagnostic accuracy, stool‐based Smart Gene™ assay removes obstacles seen in intragastric fluid testing, which requires endoscopy. Culture‐based AST is invasive and time‐consuming and often delays decision‐making regarding treatment [27]. In contrast, the Smart Gene™ stool system provides results within approximately 1 h, enabling same‐day decision‐making in outpatient settings [13]. Additionally, the Smart Gene™ stool kit is ideal for mass screening and broader clinical use, as it eliminates the need for endoscopy and physician involvement and allows patients to easily collect stool samples at home [24]. This is particularly important in Japan, where CAM‐based triple therapy is widely used as first‐line treatment under national insurance coverage, and CAM resistance is a major cause of eradication failure. Although cost‐effectiveness was not formally evaluated in this study, the ability to rapidly and noninvasively detect antibiotic resistance may reduce treatment failure and the need for repeated therapies, potentially contributing to overall healthcare cost reduction.

This study had some limitations. First, the relatively small sample size and the fact that the study was conducted at a single center limit the generalizability of the findings, as it may not fully represent the broader population affected by *H. pylori* infection. Although the results in the six patients receiving PPI/P‐CAB therapy were largely consistent with the overall diagnostic performance (see Results), the limited number of such patients precluded a formal subgroup analysis. Future studies should consider using larger sample sizes. Second, the absence of children and older participants may have failed to capture age‐related differences in diagnostic performance. Including a larger and more diverse cohort in future studies would help confirm the reliability of the results and clarify the clinical utility of stool‐based testing in a wider population range [28, 29]. Third, the possibility of misdiagnosis due to heteroresistance or mixed infections cannot be fully excluded as stool samples reflect a composite of bacterial strains from the entire gastrointestinal tract, which may differ from those detected in localized biopsy samples [19, 26]. To address this limitation, future studies should consider parallel sampling from both the stool and gastric mucosa and perform repeated testing over time to check whether the resistance results remain consistent across different sample types. Finally, this study only focused on CAM resistance. Exploring the ability of the Smart Gene™ system to detect other resistance mutations, including those related to fluoroquinolones, would help inform second‐line treatment approaches [30]. Expanding its mutation detection capabilities could further establish the system as a comprehensive diagnostic tool for managing *H. pylori* infections across various clinical treatment stages. In addition, although all analyses and interpretations were performed independently by the authors, this study was funded by the device manufacturer, and part of the laboratory procedures was supported by a company‐contracted laboratory.

## 5. Conclusions

The Smart Gene™ stool kit provides a rapid, accurate, and completely noninvasive method for diagnosing *H. pylori* infection and detecting CAM resistance, showing strong potential for broad clinical use. Its diagnostic performance is comparable to that of conventional invasive methods while offering advantages in terms of increased patient comfort, faster turnaround time, and accessibility. These features make it particularly well‐suited for use in outpatient clinics, screening programs, and situations where endoscopy is not feasible. As healthcare systems increasingly prioritize early detection and precision medicine, stool‐based molecular diagnostics such as the Smart Gene™ kit would become a key component of future *H. pylori* management strategies by enabling early detection of infection and antibiotic resistance in a noninvasive, accessible, and patient‐friendly manner. Early *H. pylori* identification allows timely eradication therapy, which is essential for preventing disease progression and reducing the long‐term risk of gastric cancer.

## Author Contributions

Momoko Tsuda performed the experiments and analyses and drafted the manuscript. Yoshiyuki Watanabe performed the pyrosequencing. Momoko Tsuda, Kimitoshi Kubo, and Mototsugu Kato performed the experiments. Mototsugu Kato supervised the study.

## Funding

Mizuho Medy Co., Ltd. provided grants and *H. pylori* molecular POCT kit for this study.

## Disclosure

In this study, the product insert draft was obtained from the manufacturer, Mizuho Medy Co., Ltd. The funder had no role in the study design, data collection and analysis, decision to publish, or preparation of the manuscript. The funder had no impact on the outcome of the clinical reports collection.

## Conflicts of Interest

The authors declare no conflicts of interest.

## Data Availability

The data that support the findings of this study are available on request from the corresponding author. The data are not publicly available due to privacy or ethical restrictions.
